# Dr. George Miller Sternberg

**Published:** 1915-12

**Authors:** 


**Dr. George Miller Sternberg,** P. S. 1860, LL.D. Michigan 1894 and Brown 1896, died at his home in Washington, November 3, aged 77. He entered the army in 1861 and was promoted until he became Surgeon General, with rank of Brigadier General, 1893, being retired in 1902. He was widely known, not only as a medical military executive, but for his personal studies in bacteriology.

